# Bilateral Thalamic Hyperintensities in a case of Viral Encephalitis

**DOI:** 10.4103/0974-777X.68541

**Published:** 2010

**Authors:** Kavitha Mohanasundaram, Srinivasan Narayanan, Subramaniyan Kumarasamy

**Affiliations:** *Department of Internal Medicine, SRM Medical College Hospital and Research Centre, SRM University, Kattankulathur, Chennai, India - 603 203*

Sir,

Japanese encephalitis is a central nervous system (CNS) infection caused by flavi virus, and it has high endemicity in southern parts of India. It is diagnosed by its clinical manifestations, imaging and serology. Thalamic hyperintensity in magnetic resonance imaging (MRI) is a specific finding which could be very useful in diagnosing Japanese encephalitis in the absence of serology. We report a case with typical MRI finding and negative serology and discuss the differential diagnosis of bilateral thalamic hyperintensities in MRI.

We admitted a 15-year-old previously healthy boy with high-grade fever and headache for three days. On the day of admission, his neurological status was clinically normal. On the second day, he developed one episode of generalized tonic-clonic seizures. Progressively he had vomiting, diplopia, slurring of speech and nasal regurgitation. On clinical examination, his mentation was normal, and there was no motor or sensory deficit, no neck stiffness and no nystagmus. His peripheral smear and quantitative buffy coat (QBC) were negative for Plasmodium falciparum infection. He was investigated for a possible CNS infection.

His cerebrospinal fluid (CSF) analysis done on the second day of admission showed mild lymphocytic pleocytosis with elevated protein. CSF glucose level was normal. MRI studies showed bilateral thalamic hyperintensity in T2-weighted Fluid-attenuated inversion-recovery *imaging* (FLAIR) [Figure 1a, b]. With this clinical scenario, a working diagnosis of viral encephalitis was made. The patient was treated with intravenous acyclovir; intravenous artesunate; ceftriaxone; intravenous methyl prednisolone; antiepileptics; and other supportive management. Results of polymerase chain reaction (PCR) tests done in CSF for herpes simplex encephalitis and tuberculosis were negative. Result of test for IgM antibodies done for Japanese encephalitis was negative. The patient was responding to the treatment initially. His vital parameters were normal. On day 7, he progressively developed acute respiratory distress syndrome (ARDS) and was put on ventilatory support. He eventually succumbed to the disease.

**Figure 1a:**
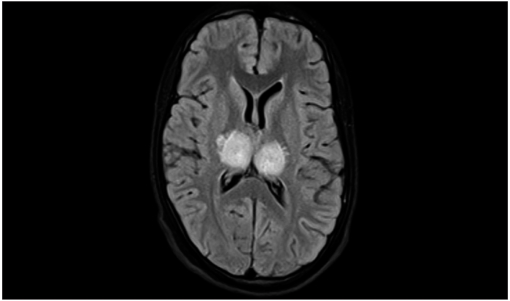
MRI T2-weighted FLAIR image showing bilateral thalamic hyperintensity

**Figure 1b:**
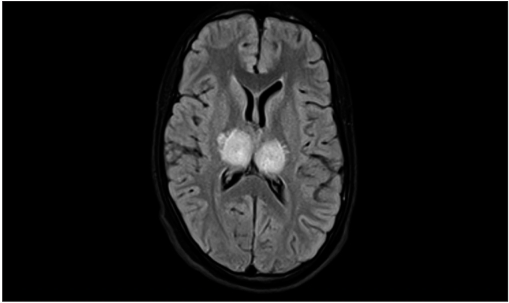
MRI T2-weighted FLAIR image showing bilateral thalamic hyperintensity

The differential diagnosis of bilateral thalamic hyperintensities in T2-weighted FLAIR images has been well studied.[1] The most common infective cause is flavivirus infection. Japanese encephalitis, West Nile and Murray virus[2] are the three viruses causing encephalitis in the group of flaviviridae. Wernicke’s encephalopathy, Wilson’s disease, cerebral venous sinus thrombosis, slow virus disease (Creutzfeldt-Jacobs), gliomas and Fabry’s disease are the other diseases that can present with similar MRI findings.

In this clinical scenario, in line with the endemicity of the disease, there was a high likelihood that this was a case of Japanese encephalitis. Dung *et al*. have reported a sensitivity of 23% and specificity of 100% for thalamic lesions in CT/ MRI for the diagnosis of Japanese encephalitis.[3] IgM antibodies in Japanese encephalitis appear after the first week of infection, which could explain the negativity of the result.

Japanese encephalitis has high prevalence in India, particularly in the southern parts. It has a peculiar biphasic pattern; however, there are only few reports available for the same. It is highly fatal, and the fatality rates can be as high as 60%.[4] The diagnosis is established by serological analysis of CSF. IgM antibodies can be detected usually on the eighth day, in some cases as early as the fourth. In endemic regions with appropriate history, even in the absence of serological evidence the most important clinical diagnosis of bilateral thalamic hyperintensities is viral encephalitis, possibly Japanese encephalitis.

## References

[1] Smith AB, Smirniotopoulos JG, Rushing EJ, Goldstein SJ (2009). Bilateral thalamic lesions. AJR Am J Roentgenol.

[2] Einsiedel L, Kat E, Ravindran J, Slavotinek J, Gordon DL (2003). MR findings in Murray Valley encephalitis. AJNR Am J Neuroradiol.

[3] Dung NM, Turtle L, Chong WK, Mai NT, Thao TT, Thuy TT (2009). An evaluation of the usefulness of neuroimaging for the diagnosis of Japanese encephalitis. J Neurol.

[4] Singh BZ, Agarwal VK (2005). Japanese encephalitis: Is routine immunization required. Med J Armed Forces India.

